# Indents

**Published:** 1920-04

**Authors:** 


### INDENTS

**☞Brief Articles of Technical or Scientific Value will receive notice and comment. Contributions invited.**

### RED CROSS NOTES

SERBIA RETURNS TO RUG MAKING. Once more Serbians are taking up the making of rugs and tapestries, which was one of their principal occupations before the war. Serbian rugs have ever been noted for the richness of their color and design as well as the durability of their dyes. Though the country has suffered much from its seven years of warfare and pillage, the knowledge of weaving and dyeing has been carefully preserved.

Serbian women have organized a school of weaving and here they work almost entirely at hand looms. Red Cross workers, who by feeding and clothing the suffering population, help them to re-establish their normal occupations, are bringing back samples of rugs and tapestries to this country. In making a market for the Serbian wares the Red Cross Commission is fulfilling its object, which includes not only the performance of relief work, but the establishment of means whereby future relief will be unnecessary.

---

A MILLION DOLLARS IN AMERICAN DRUGS. The American Red Cross gave outright $1,000,000 to purchase drugs and other medical supplies for French soldiers, during the dark days of the war, when the French Red Cross found itself unable to provide for the 780,000 wounded men lying ill in the hospitals. This is only part of the assistance rendered the French women war workers by the American organization, according to Countess d'Haussonville, a prominent French woman.

On the occasion of the closing of the wartime co-operative arrangement between the French and the American organizations the countess declared that the work of the ten thousand French women who cared for France's three-quarters of a million wounded men would have been impossible save for American aid.

---

SEDAN REVIVES BROADCLOTH INDUSTRY. Early acceleration of the broadcloth output of France is expected from the stimulation afforded the industry by the training of a number of boys for broadcloth weaving in Sedan, that city immortalized as the last triumph of the American forces before the armistice.

The project is financed by money derived from the sale of Red Cross supplies. Under the plan by which the American relief organization is assisting more than 4,000 towns in the devastated area, Red Cross articles are sold below cost to those able to buy and given to those who can not buy, and all the funds received from this source are turned over to the community to be devoted to whatever purpose the inhabitants may decide is for the general good.

In this instance the townspeople decided to revive broadcloth manufacture, which has long been one of the principal industries of Sedan, interrupted by the war.

Weaving as an industry has flourished for centuries in Sedan. The weaving of fine, black cloth was introduced by Cardinal Mazarin, famous in French history as the successor of Richelieu. It remained a staple industry until the end of the nineteenth century.

The training of the young weavers, as well as other reconstruction and relief activities promoted by the Red Cross, is under the direction of Mme. Devin, wife of a French officer, who is a trained social worker and a member of the local committee handling Red Cross supplies.

Four years of German occupation have greatly impoverished the town, the enemy having used oppressive measures to control the populace and having levied crushing taxes on the city. The first relief after the armistice came from the American Red Cross, which has continued to help the needy ever since.

---

Demand for Trained Nurses. Communities, business men, industrial operators and nurses who desire information on any phase of the Red Cross nursing situation are invited to apply to the Red Cross Bureau of Information at 44 East 23d Street, New York City, either in person or by mail. This bureau has become a national clearing house for general nursing information and the data gathered here is placed at the disposal of all who may be interested.

If a nurse desires information regarding opportunities for a future position, she is invited to apply to the bureau.

Two hundred and forty-seven American nurses, 109 of whom recently have been released from military service in this country and overseas, have been granted scholarships during the last eight months by the American Red Cross to enable them to take training in public health nursing.

Trained public health nurses are greatly in demand for the Red Cross peace-time program and in order to meet its requirements the Red Cross at the beginning of the year appropriated $100,000 to aid nurses returning from military to civilian life in fitting themselves for public health nursing.

---

Reinstate Your Lapsed U. S. Insurance. Discharged service men may reinstate their lapsed yearly renewable term insurance at any time before July 1, 1920, without regard to the date the insurance lapsed or the date of discharge from the service. The discharged man in his application for reinstatement must state that he is in as good health as at the time of his discharge or at the expiration of the grace period, whichever is the later date, and must send with the application a payment covering two monthly premiums, one to cover the grace period, one for the month of reinstatement.

This ruling of the Director of the Bureau of War Risk Insurance abrogates all previous decisions. The American Red Cross urges all discharged service men to take advantake of this new opportunity to reinstate their insurance because of the liberal features which are now included under the authorization of the Sweet Bill.

---

Clinics at the National. The prosthetic clinics, of which there were seventy-seven, were so arranged that each thirty minutes the clinics were to begin anew, thus giving every one a chance to get around. The only drawback to this system was that it didn't work, the different individuals and groups wandering around at their own sweet will for as long or as short a time as they might be interested or as long as they could see what was happening; as it ever was and ever shall be. In the prosthetic division considerable attention was given to the varied "best" methods for performing the different steps in making full dentures. In this connection, it is gratifying to know that the various authorities on this class of work have been instructed to get together and adopt a standardized and generally accepted best method. Under this section also were clinics on cast clasps, porcelain jacket crowns, carmichael and shoulder crowns, etc.—*Northwestern Journal.*

---

To Obtain Relation of Saddle to Clasp. It is known that a cast gold saddle will not go to place perfectly over the model of the case. Therefore, in a piece of work involving the use of gold saddles, the surest way to success is to obtain the relation of the saddle to the clasp or attachment under light pressure directly in the mouth. Satisfactory results may be obtained as follows: Build a modeling compound block upon the saddle to a point slightly above the occlusal plane, allowing the compound to flow around the tooth carrying the attachment. Chill and remove, slightly warm the occlusal surface of the compound over the flame and place quickly in hot water. Insert in the proper position in the mouth and allow patient to close until the opposing teeth are indented in the compound. This will equalize the pressure on the saddle. Remove and relieve the compound generously around the tooth that is to carry the attachment. Set the attachment in place. Fill in biting block with soft plaster where the compound has been cut away. Place the saddle in position and allow patient to exert slight pressure until the plaster has set. Generally when it is removed the attachment will come out with the impression, but if it should resist and become dislodged, it can be easily replaced in correct relation to the biting block. Pour a model of some investing material and solder the attachments to the saddle upon this. This method may be used in assembling two saddles at the same time, as in a lower case where we are desirous of obtaining the relationship between the saddles and clasps.—A. W. Lufkin, *Pacific Dental Gazette.*

---

CONSERVATION OR RADICALISM. The individuals who advocate complete destruction of dental organs in order to rid the body of streptococcal growth, therefore evidence profound ignorance. Wholesale extraction of teeth will not rid the body of streptococcal growth. One might as well advise removal of the transverse colon to rid the body of the colon bacillus. A much more sane method of cutting down the inroads of mouth infection is that of vigorous, systematic mouth sanitation, together with the adoption of a diet planned to prevent intestinal putrefaction.

Now, as to the other side of this question, as to when we are justified in breaking down dental mechanisms by extraction. This must rest again upon an examination of the patient's vital resistance, expressed by the blood stream, together with any evidence which tends to prove that the individual has been oversensitized or brought into a condition of anyphylaxis to streptococcal infection by a long exposure to such infection.

We need to emphasize the fact that modern means of laboratory diagnosis, and the taking of careful case histories, with the modern X-ray pictures, should be the foundation stones upon which conservative or destructive dentistry should be determined. If the dental and medical professions will get together on all cases in which extraction is advocated, and will gather and weigh the evidence each case presents, it seems altogether likely to me that we can arrive at such a sane and reasonable judgment of the conditions that justice will be done to all.—Hartzell.

---

Root Canal Technic. Conclusions of Dr. A. B. Cran:

1. No sterilization of infected teeth can be said to be complete unless it includes the canal and tubuli, the denuded cementum, and the periapical tissues.

2. The same agency can not be depended upon to complete sterilization in all these sites.

3. The canal and dentinal tubuli can probably be satisfactorily sterilized by the thorough use of sodium-potassium alloy and thirty per cent. sulphuric acid, followed by dressings of mild antiseptic oils; or by Howe's silver reduction method.

4. The periapical region may be sterilized by inducing the inflammatory reaction by means of electrolytic medication, followed by immediate and sufficient control.

5. The apical cementum can at best receive but superficial sterilization, and unless the denuded portion is small enough to be successfully capped with gutta-percha it must be surgically removed.

6. The establishment of scientifically correct culture methods and media is the crying need of the moment. Until this is accomplished the filling of the canal must be a probationary expedient.—March *Cosmos.*

---

Toothsome but not Cheering. The American Academy of Dental Science no more than crystallizes a doctrine that has been in process of development for some time when it condemns the removal of nerves from decayed teeth as a preparation for the insertion of fillings, presumably because it is convinced that the practice makes room for dangerous abscesses. And though its announcement that gold in the mouth is unsanitary is rather startling, it neglects to endorse the extreme position of those iconoclasts—we believe the word is used advisedly—who would have all bridgework eliminated on the premise that the strain put thereby on masticating apparatus is excessive and causes all sorts of hidden festers which give rise in turn to rheumatism, heart trouble and goodness knows what other ills.

Nevertheless the academy goes far enough to make a reader's molars and incisors exceedingly jumpy; and if we are to believe all that is being told us by the alarmists, most of the recent advance in dental science has been no advance at all, but on the contrary has been merely mistaken experimentation. Consequently, those of us unfortunate enough to be cursed with the degenerate ivories of civilization are condemned henceforth to the old-fashioned store teeth on plates or to the acquisition of the gentle art of "gumming it." Though, of course, there may be some who will decide that to go crazy from a blind abscess is no worse than to do so from the irritation of a rubber roof.

The principal comfort one finds in the dilemma the dentists have created is the thought that the extreme reactionaries and pessimists of the profession may be as far wrong as were the cheerful optimists and propagandists of a few years ago who looked upon root filling and the installation of bridgework as the only means of human salvation.—*Detroit Free Press.*

